# Supplementary material for: A powerful drug combination strategy targeting glutamine addiction for the treatment of human liver cancer
Source: eLife. 2020 Oct 5;9:e56749. doi: 10.7554/eLife.56749 (PMC7535927; doi:10.7554/eLife.56749)
Supplement: Figure 3—source data 2. [file elife-56749-fig3-data2.docx]

| **Drug** | **Target** | **SNU398** | | **HepG2** | |
| --- | --- | --- | --- | --- | --- |
|  |  | IC50  (μM) | Enhance  （%） | IC50  (μM) | Enhance  （%） |
| BAY-876 | GLUT1 | 4.085 | 1.5 | >100 | 1.9 |
| AZD3965 | MCT1 | 0.788 | 0.1 | >100 | -0.1 |
| CPI613 | PDH/α-KGDH | >100 | 7.3 | >100 | 3.3 |
| Compound 3k | PKM2 | 0.237 | -4.7 | 15.82 | -6.7 |
| NCT503 | PHGDH | 28.35 | 7.1 | 37.09 | 5.9 |
| ND-646 | ACC1/ACC2 | 11.59 | 0.9 | 50.85 | 0.7 |
| AG221 | IDH2 | 13.64 | 13.7 | 36.39 | 16.3 |
| NLG-8189 | IDO | >100 | 1.4 | >100 | 2.3 |
| IACS-10759 | OXPHOS | 10.83 | 7.7 | 15.75 | 12.8 |
| Dapagliflozin | SGLT2 | 65.33 | -3.8 | 87.88 | 5.8 |
| 2-DG | HK2 | 548.8 | 3.7 | 14.04  (mM) | 0.6 |
| V-9302 | ASCT2 | 8.402 | 20.3 | 19.25 | 31.6 |
| DHEA | PPP | 97.01 | 11.7 | >100 | 11.2 |
