# Supplementary material for: A powerful drug combination strategy targeting glutamine addiction for the treatment of human liver cancer
Source: eLife. 2020 Oct 5;9:e56749. doi: 10.7554/eLife.56749 (PMC7535927; doi:10.7554/eLife.56749)
Supplement: Transparent reporting form [file elife-56749-transrepform.docx]

***eLife’s* transparent reporting form**

We encourage authors to provide detailed information *within their submission* to facilitate the interpretation and replication of experiments. Authors can upload supporting documentation to indicate the use of appropriate reporting guidelines for health-related research (see [EQUATOR Network](http://www.equator-network.org/%20)), life science research (see the [BioSharing Information Resource](https://biosharing.org/)), or the [ARRIVE guidelines](http://www.plosbiology.org/article/info:doi/10.1371/journal.pbio.1000412) for reporting work involving animal research. Where applicable, authors should refer to any relevant reporting standards documents in this form.

If you have any questions, please consult our Journal Policies and/or contact us: [editorial@elifesciences.org](mailto:editorial@elifesciences.org).

**Sample-size estimation**

- You should state whether an appropriate sample size was computed when the study was being designed
- You should state the statistical method of sample size computation and any required assumptions
- If no explicit power analysis was used, you should describe how you decided what sample (replicate) size (number) to use

Please outline where this information can be found within the submission (e.g., sections or figure legends), or explain why this information doesn’t apply to your submission:

At the time of the design of the experiments, the study was largely exploratory and effect size was unknown, meaning that sample size / power estimation was not feasible.

**Replicates**

- You should report how often each experiment was performed
- You should include a definition of biological versus technical replication
- The data obtained should be provided and sufficient information should be provided to indicate the number of independent biological and/or technical replicates
- If you encountered any outliers, you should describe how these were handled
- Criteria for exclusion/inclusion of data should be clearly stated
- High-throughput sequence data should be uploaded before submission, with a private link for reviewers provided (these are available from both GEO and ArrayExpress)

Please outline where this information can be found within the submission (e.g., sections or figure legends), or explain why this information doesn’t apply to your submission:

Long-term colony formation assays were performed at least 2 times (2 biological replicates)；Incucyte cell proliferation assay and apoptosis assay were performed within at least 3 technical replicates. Metabolomics analyses were performed with 3 biological replicates. Western blots were performed at least 2 times (2 biological replicates).

Biological replicate: These were the independent experiments, the same test/experiment was performed using a different batch of cells at a different time. Technical replicate: testing the same sample (biological replicate) multiple times (3 or more) within an experiment.

**Statistical reporting**

- Statistical analysis methods should be described and justified
- Raw data should be presented in figures whenever informative to do so (typically when N per group is less than 10)
- For each experiment, you should identify the statistical tests used, exact values of N, definitions of center, methods of multiple test correction, and dispersion and precision measures (e.g., mean, median, SD, SEM, confidence intervals; and, for the major substantive results, a measure of effect size (e.g., Pearson's r, Cohen's d)
- Report exact p-values wherever possible alongside the summary statistics and 95% confidence intervals. These should be reported for all key questions and not only when the p-value is less than 0.05.

Please outline where this information can be found within the submission (e.g., sections or figure legends), or explain why this information doesn’t apply to your submission:

Figure 1d and 1e, GSEA plots were generated using javaGSEA using the default settings resulting in indicated p-values. Figure 1f , Figure 2c, Figure 2e and Figure 2f, p values were derived by the Kaplan-Meier log rank test.

For Figure 2a, Figure 3b, Figure 4a, Figure 5a, Figure 5e, Figure 5f, Figure 6a-d, Figure 6g-l, error bars represent the average data of at least 3 independent experiments (biological replicates) using 3 technical replicates within each experiment (± SEM). P-values are indicated with ***P < 0.001, **P < 0.01 and *P < 0.05 (two-tailed t-test).

(For large datasets, or papers with a very large number of statistical tests, you may upload a single table file with tests, Ns, etc., with reference to sections in the manuscript.)

**Group allocation**

- Indicate how samples were allocated into experimental groups (in the case of clinical studies, please specify allocation to treatment method); if randomization was used, please also state if restricted randomization was applied
- Indicate if masking was used during group allocation, data collection and/or data analysis

Please outline where this information can be found within the submission (e.g., sections or figure legends), or explain why this information doesn’t apply to your submission:

n/a

**Additional data files (“source data”)**

- We encourage you to upload relevant additional data files, such as numerical data that are represented as a graph in a figure, or as a summary table
- Where provided, these should be in the most useful format, and they can be uploaded as “Source data” files linked to a main figure or table
- Include model definition files including the full list of parameters used
- Include code used for data analysis (e.g., R, MatLab)
- Avoid stating that data files are “available upon request”

Please indicate the figures or tables for which source data files have been provided:

Figure 1-source data 1 Liver cancer is addicted to glutamine；

Figure 2-source data 1 The glutaminase inhibitor CB-839 monotherapy shows insufficient anti-tumor effect in liver cancer；

Figure 3-source data 1 A compounds screen identifies ASCT-2 inhibitor V-9302 sensitizing GD liver cancer cells to CB-839 treatment；

Figure 3-source data 2 Data related to Figure 3d;

Figure 4-source data 1 Combination of CB-839 and V-9302 shows potential synergy in multiple GD liver cancer cells；

Figure 5-source data 1 Combination of CB-839 and V-9302 depletes GSH and induces lethal ROS level in GD liver cancer cells；

Figure 6-source data 1 Combined treatment inhibits xenograft growth and induces apoptosis *in vivo.*
